# The Delivery of the Recombinant Protein Cocktail Identified by Stem Cell-Derived Secretome Analysis Accelerates Kidney Repair After Renal Ischemia-Reperfusion Injury

**DOI:** 10.3389/fbioe.2022.848679

**Published:** 2022-05-11

**Authors:** Ji Hyun Kim, Heejo Yang, Michael W. Kim, Kang Su Cho, Doo Sang Kim, Hyung Eun Yim, Zachary Atala, In Kap Ko, James J. Yoo

**Affiliations:** ^1^ Wake Forest Institute for Regenerative Medicine, Wake Forest School of Medicine, Winston-Salem, NC, United States; ^2^ Department of Urology, Soonchunhyang University College of Medicine, Cheonan, South Korea; ^3^ Department of Urology, Gangnam Severance Hospital, Yonsei University College of Medicine, Seoul, South Korea; ^4^ Department of Pediatrics, Korea University College of Medicine, Seoul, South Korea

**Keywords:** stem cell secretome, conditioned medium, recombinant protein cocktail, kidney disease, tissue engineering and regenerative medicine

## Abstract

Recent advances in cell therapy have shown the potential to treat kidney diseases. As the treatment effects of the cell therapies are mainly attributed to secretomes released from the transplanted cells, the delivery of secretomes or conditioned medium (CM) has emerged as a promising treatment option for kidney disease. We previously demonstrated that the controlled delivery of human placental stem cells (hPSC)-derived CM using platelet-rich plasma (PRP) ameliorated renal damages and restored kidney function in an acute kidney injury (AKI) model in rats. The proteomics study of the hPSC-CM revealed that hPSC secrets several proteins that contribute to kidney tissue repair. Based on our results, this study proposed that the proteins expressed in the hPSC-CM and effective for kidney repair could be used as a recombinant protein cocktail to treat kidney diseases as an alternative to CM. In this study, we analyzed the secretome profile of hPSC-CM and identified five proteins (follistatin, uPAR, ANGPLT4, HGF, VEGF) that promote kidney repair. We investigated the feasibility of delivering the recombinant protein cocktail to improve structural and functional recovery after AKI. The pro-proliferative and anti-apoptotic effects of the protein cocktail on renal cells are demonstrated *in vitro* and *in vivo*. The intrarenal delivery of these proteins with PRP ameliorates the renal tubular damage and improved renal function in the AKI-induced rats, yielding similar therapeutic effects compared to the CM delivery. These results indicate that our strategy may provide a therapeutic solution to many challenges associated with kidney repair resulting from the lack of suitable off-the-shelf regenerative medicine products.

## Introduction

Acute kidney injury (AKI) is a common complication in critically ill patients, resulting in a rapid decline in renal function. With a high mortality rate exceeding 50%, the treatment of AKI remains a challenge (Griffin et al., 2020). The only treatment options for patients with AKI include dialysis and supportive care using preventive or pharmacotherapeutic drugs; however, these treatments have shown limited clinical efficacy and further kidney deterioration (Patschan and Muller, 2015). AKI often progresses to chronic kidney disease (CKD) and end-stage renal disease (ESRD), requiring renal replacement therapy such as life-long hemodialysis and kidney transplantation.

Recent advances in cell therapies offer promising therapeutic options for treating kidney diseases (Yamaleyeva et al., 2012; Stenvinkel et al., 2016; Kim et al., 2020). However, safety concerns remain a critical issue for clinical practices of the cell transplantation approach, including immune rejection, pulmonary embolism, and teratoma formation (Herberts et al., 2011). Low efficacy due to the poor engraftment and survival of the administered cells are another issue (Baldari et al., 2017). In addition, several reports have demonstrated that the treatment effects of the cell therapy are mainly attributed to trophic factors secreted by the cells (secretomes) rather than the tissue-specific differentiation and tissue formation of the transplanted cells (Srijaya et al., 2014; Lukomska et al., 2019; Yim et al., 2019).

As an alternative approach to cell therapies, the treatment using stem cell-derived secretomes has been investigated, showing great potential in tissue repair. Secretomes can be used as a form of conditioned medium (CM). The CM contains soluble proteins (growth factors and cytokines) and extracellular vesicles (EV) that promote tissue repair associated with proliferation, anti-apoptosis, anti-inflammation, immunomodulation, and pro-angiogenesis (Gnecchi et al., 2008; Baraniak and McDevitt, 2010; Pawitan, 2014). The therapeutic effects of the exogenous delivery of CM on improving tissue regeneration have been demonstrated (Fukuoka et al., 2012; Zhou B.-R. et al., 2013; Pawitan, 2014; Katagiri et al., 2016; Katagiri et al., 2017; Almeria et al., 2019; Palama et al., 2020). Also, several studies have shown the reno-protective effects of CM derived from mesenchymal stem cells (MSC), endothelial progenitor cells (EPC), induced pluripotent stem cells (iPSC), and human placental stem cells (hPSC) (Cantaluppi et al., 2015; Abedi et al., 2016; Nassar et al., 2016; Tarng et al., 2016; Yim et al., 2019). However, rapid diffusion and degradation of the administrated CM in the body reduces the therapeutic efficacy and fails functional recovery in a damaged kidney (Pawitan, 2014; Abedi et al., 2016; Tarng et al., 2016).

Our previous study developed a novel strategy of controlled delivery of CM derived from human placenta-derived stem cells (hPSC-CM) to treat kidney diseases (Yim et al., 2019). hPSCs have recently been used as a valuable resource for tissue regeneration with multipotent, immunosuppressive, anti-apoptotic, and anti-oxidative properties (Hao et al., 2000; Liu et al., 2010; Oliveira and Barreto-Filho, 2015). In addition, our previous study showed that the hPSC secreted a large number of proteins related to cell survival and angiogenesis in significantly higher abundance compared with amniotic fluid stem cells (AFSC), adipose-derived stem cells (ADSC), and human umbilical vein endothelial cells (HUVEC) (Matz et al., 2020). We confirmed the pro-proliferative and anti-apoptotic effects of hPSC-CM treatment on primary human renal tubular cells (hRC) *in vitro*. The intrarenal injection of hPSC-CM ameliorated renal damage in a renal ischemia/reperfusion (I/R)-induced AKI model in rats showed similar therapeutic effects to the HPSC transplantation. To further enhance the hPSC-CM treatment efficiency, we utilized platelet-rich plasma (PRP) gel capable of controlled delivery and sustained release of CM in the damaged kidney. The controlled delivery of hPSC-CM using the PRP system into the I/R-induced kidney prevented renal cell apoptosis and tubular damage, leading to renal function improvement with decreased serum creatinine level (Yim et al., 2019).

While the controlled delivery of hPSC-CM demonstrated promising outcomes for AKI treatment, several challenges remain to be addressed for clinical translation and commercialization. The major issues include inevitable massive cell culture for CM production, inconsistency of product quality, and the presence of unidentified or unknown components in CM (Tran and Damaser, 2015). In several studies using CM, the precise mechanism by which the secreted factors are effective for tissue repair remains unknown (Tran and Damaser, 2015).

To better understand the potential mechanism involved in the therapeutic effects of hPSC-CM, we performed quantitative protein analyses. In our previous study, we found that the hPSC-CM contains several proteins that can promote kidney repair and regeneration, such as follistatin, urokinase-type plasminogen activator receptor (uPAR), angiopoietin-like 4 (ANGPTL4), hepatocyte growth factor (HGF), and vascular endothelial growth factor (VEGF). This result suggested that these proteins are the main factors contributing to the attenuation of the I/R-induced renal injury (Yim et al., 2019).

Taken together, this study hypothesizes that the specific proteins identified by hPSC-CM secretome analysis and promote kidney repair can be used to treat kidney diseases as a form of recombinant protein cocktail (RPC) instead of using an unidentified secretome composition as a whole in CM. The controlled delivery of RPC into I/R-induced kidney using the PRP system will increase renal cell survival and proliferation, attenuate renal injuries and ameliorate renal function *in vivo*. We expect that the combination of these recombinant proteins could replace the function of CM and achieve similar therapeutic effects to that observed in the CM treatment, thereby addressing the several concerns raised on the CM treatment above by using a combination of commercially available recombinant proteins.

To demonstrate the hypothesis, we analyze the secretome profile of hPSC-CM and identify proteins effective for kidney repair. A specific combination of recombinant proteins (RPC) is developed based on the identified proteins and their concentrations. We investigate the feasibility of delivering RPC with the PRP system to achieve structural and functional recovery after AKI. The present study focuses on demonstrating the effects of RPC delivery on attenuating renal tubular damages by enhancing hRC survival and proliferation. To confirm the effectiveness of the combination of proteins, hRC proliferation and anti-apoptosis of the RPC treatment are compared with those of each protein and hPSC-CM. The therapeutic effect of intrarenal delivery of RPC with PRP on renal injury repair is demonstrated using the rat I/R-induced AKI model.

## Materials and Methods

### Preparation of Human Placental Stem Cells-Conditioned Medium and Recombinant Protein Cocktail

**FIGURE 1:**
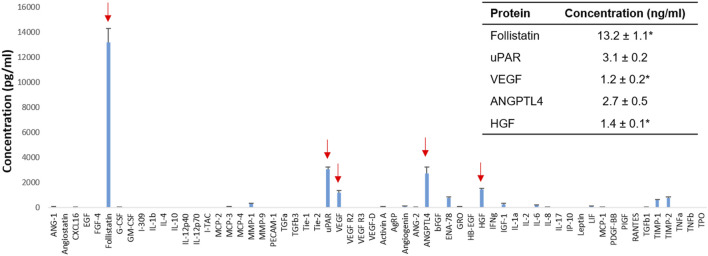
Human placental stem cell (hPSC)-derived secretome analysis. Identification of human proteins that promote kidney repair and regeneration (arrows) and their concentrations in the 4× concentrated HPSC-derived conditioned media (CM) (*n* = 4). *MAX (value above the highest standards). ANGPTL4: Angiopoietin-like four; HGF: Hepatocyte growth factor; uPAR: Urokinase receptor; VEGF: Vascular endothelial growth factor.

hPSC was obtained from the Regenerative Medicine Clinical Center at the Wake Forest Institute for Regenerative Medicine (Zhang et al., 2016; Yim et al., 2019). hPSCs were cultured in growth media (GM) composed of 15% fetal bovine serum (FBS; Gibco, Carlsbad, CA), 18% Chang B (Irvine Scientific, Santa Ana, CA), 2% Chang C (Irvine Scientific), 1% L-glutamine, and 1% penicillin-streptomycin (PS; GE Healthcare Life Sciences, Logan, UT) in Dulbecco’s modified Eagle medium (DMEM)/low-glucose (GE Healthcare Life Sciences). The medium was changed every 2–3 days. The hPSCs (passages 10–13, 1.5 cells × 10^6^ cells) were plated in a 15 cm culture dish and incubated in GM until the cells reached 60% confluence and then cultured in serum-free media (SFM; without FBS and Chang C) for an additional 3 days. CM was collected and filtered through a 0.22 μM filter. The CM was concentrated to 4-fold (4×) using a centrifugal filter unit (3 kDa, Merck Millipore, Darmstadt, Germany) (Yim et al., 2019). For proteomics, human proteins within the hPSC-CM were analyzed (*n* = 4) (Quantibody^®^ quantitative multiplex ELISA array, Raybiotech, Norcross, GA). For *in vitro* and animal studies, 4× concentrated CM in SFM was used. The RPC composed of recombinant human follistatin (13.2 ng/ml), HGF (1.4 ng/ml), VEGF (1.2 ng/ml), uPAR (3.1 ng/ml), and ANGPTL4 (2.7 ng/ml) (R&D systems, Minneapolis, MN) was prepared (Figure 1). The concentration of CM (4×) was optimized and determined in our previous study (Yim et al., 2019). The concentration of each protein was determined based on the results of the 4× concentrated hPSC-CM analysis, which was the same as that in the 4× CM.

### *In Vitro* Experiments

hRCs were isolated and cultured using the established protocol in our laboratory (George et al., 2016; Yim et al., 2019). The hRCs were cultured in GM containing a mixture of two types of media (1:1). One is DMEM/High glucose containing 10% FBS and 1% PS, and the other is keratinocyte medium (Thermo Fisher Scientific, Rochester, NY) supplemented with bovine pituitary extract, epidermal growth factor, 1% FBS, 0.08% insulin-transferrin-selenium (ITS; Lonza, Basel, Switzerland), and 1% PS. hRCs were seeded in 48-well plates (5,000 cells per well) and incubated in hypoxia (1% O_2_) or normoxia (21% O_2_) for 3 days with GM (positive control), SFM (negative control), CM (4×), single protein (ANGPTL4, follistatin, HGF, uPAR, or VEGF) or RPC (ANGPTL4 + follistatin + HGF + uPAR + VEGF) (Figure 1). For the normoxic culture, cells were cultured in a regular cell culture incubator with 21% O_2_ and 5% CO_2_. For the hypoxic culture, seeded cells were transferred in a hypoxia C-chamber (BioSpherix, Parish, NY) inside a standard CO_2_ incubator (Revco Ultima II; Thermo Fisher Scientific) with a compact gas oxygen controller (ProOx P110; BioSpherix) to maintain oxygen concentration at 1 and 5% of CO_2_ (Yim et al., 2019).

### *In Vitro* Evaluations

Cells were fixed with 4% paraformaldehyde at room temperature (RT) for 10 min and permeabilized with 3% ice-cold methanol at −20°C for 10 min. The samples were incubated with serum-free protein block (Agilent Dako, Santa Clara, CA) for 15 min at RT. Primary antibodies, proliferating cell nuclear antigen (PCNA, 1:100 dilution; Abcam, Cambridge, UK), cleaved caspase-3 (1:200; Cell Signaling Technology, Beverly, MA), or aquaporin-1 (AQP-1, 1:500; Abcam), were applied respectively and incubated overnight at 4°C or for 1 h at RT. The samples were incubated with biotinylated goat anti-rabbit IgG antibody (1:200; Vector Laboratories, Burlingame, CA) for 30 min, then streptavidin-conjugated with AF594 (1:200, Thermo Fisher Scientific) incubation for 20 min at RT. DAPI (1:1000; Thermo Fisher Scientific) was applied and incubated for 10 min for a nuclear counterstain. The samples were washed with phosphate-buffered saline (PBS) three times at 5-minute intervals for each step. Antibodies were diluted with antibody diluent (Agilent Dako), and DAPI was diluted with PBS (Yim et al., 2019). For the quantification, the cells were imaged using fluorescence microscopy (DM4000, Image Pro 6.3., cellSens Dimension 1.18, Leica), and the percent of positive cells relative to the total cell number was calculated. Cell viability was evaluated using a live/dead viability kit (Invitrogen, Carlsbad, CA) according to the manufacturer’s instruction. Briefly, cells were incubated in a mixture of calcein-AM (4 mm) and ethidium homodimer-1 (2 mm) at RT for 10–20 min. The live cells (green) and dead cells (red) were visualized by fluorescence microscopy (DM4000). The number of live and dead cells was manually counted, and the cell viability (%) was calculated.

### Animal Experiments

The animal research protocol was approved by the Institutional Animal Care and Use Committee of the Wake Forest School of Medicine. All surgical procedures were performed according to the instructions provided by the Institute for Laboratory Animal Research Guide for the Care and Use of Laboratory Animals. AKI was induced by I/R in rats, and the treatment effects were evaluated for 7 days followed by injecting saline, CM + PRP, or CMF + PRP (mortality rate: 18%) (Yamaleyeva et al., 2012; Yim et al., 2019). Nude rats (male, 250–350 g, Charles River Lab, Wilmington, MA) were anesthetized by intraperitoneal injection of sodium pentobarbital (30—50 mg/kg). The renal arteries and veins of both kidneys were obstructed using non-traumatic vascular clamps. After 60 min, the clamps were removed, and reperfusion of the kidneys was visually confirmed. The rats (total nine rats) were randomly divided into three groups: 1) saline (no treatment) (*n* = 3), CM + PRP (*n* = 3), and 3) RPC + PRP (*n* = 3) injection groups. Immediately after inducing the I/R injuries, 200 μl of saline, CM + PRP, or RPC + PRP was injected into the upper and lower poles of each kidney (50 μl per site). Age-matched control (normal, without I/R injuries; *n* = 3) was included in this study. The PRP was obtained from porcine blood using the established method in our laboratory (Landesberg et al., 2005; Yim et al., 2019). Porcine blood (Lumpire Biological Laboratories, Pipersville, PA) was centrifuged at 400 g for 10 min. Plasma and buffy coat in the supernatant were collected and centrifuged at 800 g for 10 min. A supernatant PRP layer was collected. For the CM + PRP injection, 200 μl of 4× CM with PRP was finally made with a mixture of PRP (20 μl, 400 × 10^6^ platelets), thrombin (40 U/ml, 100 μl, Sigma Aldrich, St. Louis, MO) and 10× concentrated CM (80 μl). For the RPC + PRP injection, the five proteins reconstituted in saline (80 μl) were mixed with 20 μl of PRP and 100 μl of thrombin. The final concentration of the follistatin (13.2 ng/ml), HGF (1.4 ng/ml), VEGF (1.2 ng/ml), uPAR (3.1 ng/ml), and ANGPTL4 (2.7 ng/ml) of the CM + PRP and RPC + PRP group was the same (Yim et al., 2019).

### *In Vivo* Evaluations

Blood samples were collected from saphenous veins at 0 (before I/R) and 1, 2, 3, and 7 days after I/R and injections. Serum creatinine concentrations (mg/dl) were measured using the Creatinine assay kit (Sigma Aldrich). The serum creatinine concentrations at each time point were divided by that at day 0 to obtain normalized serum creatinine value. After 7 days, kidneys were collected and fixed with 4% paraformaldehyde overnight. Paraffin-embedded sections (5 μM) of renal tissues were stained with Hematoxylin and Eosin (H&E) and Periodic acid-Schiff (PAS) staining using standard protocols. The level of acute tubular injury was evaluated using a semi-quantitative scoring system (0–4) by two blinded observers (*n* = 3 per group and nine random fields per sample (total 27 fields per group). The percent of total cortical tubules showing tubular cast formation, tubular dilatation, and tubular degeneration (loss of brush border, detachment of tubular epithelial cells, vacuolar change, and condensation of tubular nuclei) was estimated with the following criteria under optical microscopes: 0, normal; 1, < 25%; 2, 25–50%; 3, 50–75%; 4, > 75% (Yim et al., 2019). Cell proliferation and apoptosis were evaluated with immunofluorescent staining for PCNA and cleaved caspase-3. Deparaffinized tissue sections were headed in 0.01 M citrated buffer (pH 6.0, Polysciences, Warminster, PA) for 20 min. Immunofluorescent staining for PCNA and cleaved caspase-3 was performed as described above. The percentage of PCNA- and cleaved caspase-3-positive tubular cells was measured by two blind observers (*n* = 3 per group, five random fields per sample (total 15 fields per group).

### Statistical Analysis

Results were analyzed with Origin Pro 8.5 (OriginLab Co., Northampton, MA) and SPSS software (SPSS, version 19; IBM, Armonk, NY). One-way or two-way analysis of variance (ANOVA), Tukey’s *post hoc* tests, was applied to mean comparisons. Data are presented as a mean ± standard deviation. Error bars represent standard deviation. Differences between experimental groups were considered statistically significant at *p <* 0.05.

## Results

### Human Placental Stem Cells-Derived Secretome Analysis

The secretome profile of hPSC was obtained by protein analysis of hPSC-CM (*n* = 4) (Figure 1). The 4× concentrated hPSC-CM contained several proteins that promote kidney repair and regeneration *in vitro* and *in vivo* and were expressed in higher concentrations than normal physiological serum levels. For example, follistatin (13.2 ± 1.1 ng/ml in 4× hPSC-CM) protects renal tubules from ischemia-induced apoptosis and promotes renal tubule regeneration (Maeshima et al., 2001). uPAR (3.1 ± 0.2 ng/ml) attenuates renal fibrosis and reduces renal cell apoptosis (Zhang et al., 2003a; Zhang et al., 2003b). VEGF (1.2 ± 0.2 ng/ml) promotes renal cell survival and proliferation (Villegas et al., 2005). ANGPTL4 (2.7 ± 0.5 ng/ml) reduces renal injury and proteinuria and promotes kidney vascularization in ischemia (Le Jan et al., 2003; Chugh et al., 2014; Clement et al., 2014). HGF (1.4 ± 0.1 ng/ml) prevents apoptosis of renal tubular cells and ameliorates initial injury after I/R (Miller et al., 1994; Liu et al., 1998; Liu, 1999).

In addition, some proteins effective for renal protection and recovery were detected in the hPSC-CM; however, their concentrations were lower than physiological concentrations, such as MMP-1 (308.7 ± 27.7 pg/ml), Angiogenin (106.5 ± 5.4 pg/ml), and IGF-1 (268.2 ± 88.7 ng/ml). MMP-1 inhibits renal fibrosis by regulating the degradation of extracellular matrices (Nakamura et al., 2000; Genovese et al., 2014). Angiogenin promotes neovascularization and delays the progression of kidney diseases (Tello-Montoliu et al., 2006; Anderson et al., 2018). IGF-1 reduces glomerular and tubular cell apoptosis after I/R injury and accelerates the recovery of the glomerular filtration rate (Zhu et al., 2017; Haffner et al., 2021).

Also, proteins that have a negative impact on kidney repair were detected, such as MCP-3 (97.1 ± 6.6 pg/ml), ENA-78 (781.1 ± 79.8 pg/ml), IL-6 (205.0 ± 15.0 pg/ml), TIMP-1 (610.0 ± 26.9 pg/ml), and TIMP-2 (757.7 ± 107.5 pg/ml). For instance, MCP-3 and IL-6 cause injurious inflammation in AKI (Nechemia-Arbely et al., 2008; Jones et al., 2015; Wang et al., 2021). ENA-78, TIMP-1, and TIMP-2 induce renal fibrosis (Genovese et al., 2014; Riedel et al., 2016).

Based on the analysis of the secretome profile and the literature study, we selected five proteins, follistatin, uPAR, VEGF, ANGPLT4, and HGF. These proteins were selected based on two criteria—1) effective for kidney repair and regeneration and 2) expressed in the hPSC-CM higher than the physiological concentration. The inhibitory proteins on kidney repair were excluded (e.g., MCP-3, ENA-78, IL-6, TIMP-1, TIMP-2). The proteins known to contribute to kidney repair but expressed in lower levels than the physiological serum concentrations were not included (e.g., MMP-1, Angiogenin, IGF-1). With the defined dosage of the selected proteins, we developed a specific protein combination using commercial recombinant proteins (RPC) for the application for kidney repair.

### The Effects of Recombinant Protein Cocktail on Human Renal Tubular Cells Proliferation and Anti-apoptosis—Single Protein vs. Combinatorial Proteins (Recombinant Protein Cocktail)

**FIGURE 2:**
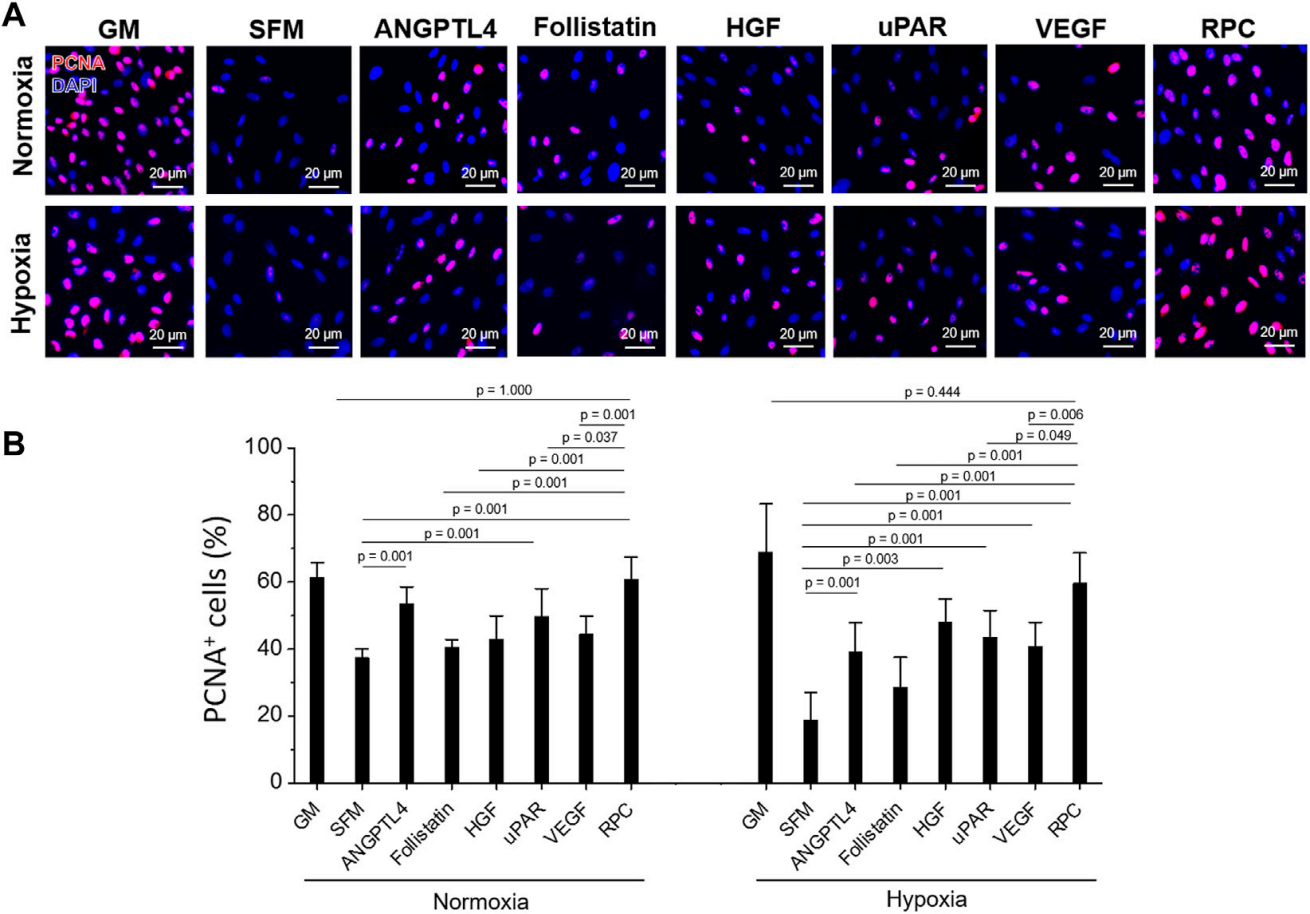
Human primary renal cell (hRC) proliferation - Single protein vs. combinatorial proteins. Treatment to hRCs with single protein (ANGPTL4, follistatin, HGF, uPAR, or VEGF) or their combination (recombinant protein cocktail (RPC)) for 3-day culture under normoxic (21% O_2_) and hypoxic (1% O_2_) conditions. **(A)** Immunofluorescence for proliferating cell nuclear antigen (PCNA, red)/DAPI (blue). **(B)** Quantification of PCNA^+^ cells (%) (*n* = 8 per group, one-way ANOVA and Tukey’s test). Scale bars: 20 µM. GM: growth medium. PCNA: proliferating cell nuclear antigen. SFM: serum-free medium.

**FIGURE 3:**
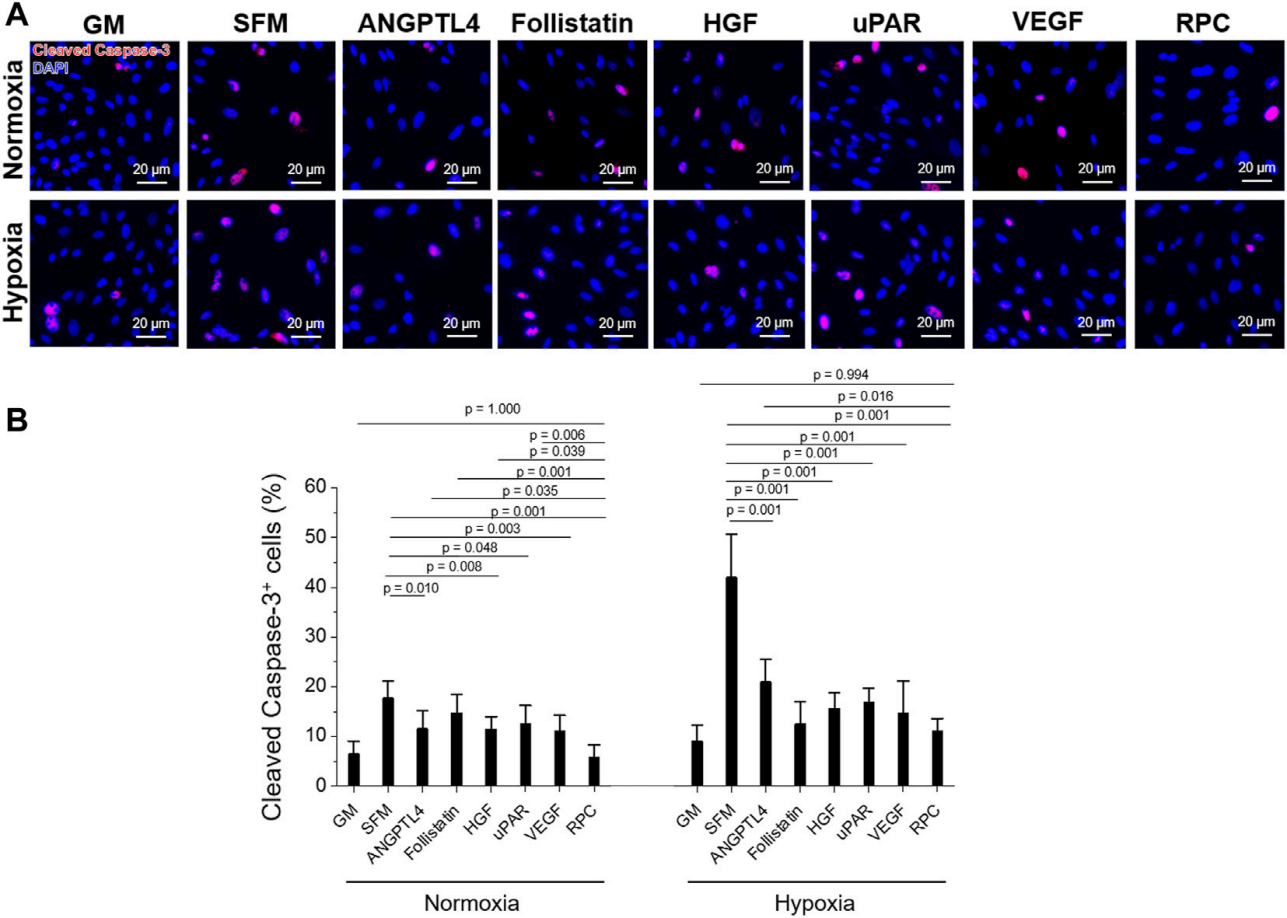
hRC apoptosis—Single protein vs. combinatorial proteins. Treatment with single protein or their combination (RPC) for 3-day culture under normoxic and hypoxic conditions. **(A)** Immunofluorescence for cleaved caspase-3 (red)/DAPI (blue). **(B)** Quantification of cleaved caspase-3^+^ cells (%) (*n* = 8 per group, one-way ANOVA and Tukey’s test). Scale bars: 20 µM.

To confirm the effectiveness of RPC, cell proliferation and anti-apoptosis of a single protein (ANGPTL4, Follistatin, HGF, uPAR, VEGF) and a combination of the five proteins (RPC) were tested with hRCs cultured in normoxic (21% O_2_) and hypoxic (1% O_2_) conditions (*n* = 8 per group, one-way ANOVA and Tukey’s test) (Figures 2, 3). To simulate the cellular behaviors under the I/R-induced kidney *in vitro*, hRCs were cultured under the hypoxic condition (Yim et al., 2019). The quantification result of PCNA^+^ cells (%), ANGPTL4, uPAR, and RPC groups showed a significant increase in hRC proliferation compared with the SFM group in the normoxic condition (*p* = 0.001). The RPC group showed a significant increase in hRC proliferation compared with the uPAR group (*p* = 0.037) (Figure 2B). In hypoxic conditions, there was an increase in PCNA^+^ hRCs (%) in ANGPTL4 (*p* = 0.001), HGF (*p* = 0.003), uPAR (*p* = 0.001), VEGF (*p* = 0.001), and RPC (*p* = 0.001) groups compared with the SFM group. The RPC group showed an increased hRC proliferation compared with ANGPTL4 (*p* = 0.001), follistatin (*p* = 0.001), uPAR (*p* = 0.049), and VEGF (*p* = 0.006) groups (Figure 2B). Only the RPC group showed no significant difference in hRC proliferation compared with the GM group in both normoxic (*p* = 1.000) and hypoxic (*p* = 0.444) culture conditions.

hRCs apoptosis treated with ANGPTL4 (*p* = 0.010), HGF (*p* = 0.008), uPAR (*p* = 0.048), VEGF (*p* = 0.003), and RPC (*p* = 0.001) was significantly decreased under normoxic conditions, showing decreased cleaved caspase-3^+^ cells (%) compared with the SFM group (Figure 3B). The RPC group showed a significant decrease in cell apoptosis compared with ANGPTL4 (*p* = 0.035), follistatin (*p* = 0.001), HGF (*p* = 0.039), and uPAR (*p* = 0.006) groups. In hypoxic conditions, the anti-apoptotic effect was demonstrated from all individual proteins and RPC groups compared with the SFM group (*p* = 0.001), and the RPC group showed a higher anti-apoptosis effect than the ANGPTL4 group (*p* = 0.016). In both normoxic and hypoxic conditions, only the RPC treated group showed no significant difference in cell apoptosis with the GM group (*p* = 0.994). The cell number of the single protein and RPC groups was higher than SFM in normoxic and hypoxic (1% O_2_) conditions (*n* = 3 per group, one-way ANOVA and Tukey’s test) (Supplementary Figure S1). Overall, the RPC group demonstrated higher proliferative and anti-apoptosis effects than the single protein groups and SFM and showed a similar percent of proliferation and apoptosis as the GM group in both hypoxic and normoxic conditions. These results confirmed that the combination of proteins (RPC) is more effective in hRC proliferation and anti-apoptosis than individual proteins.

### The Effects of Recombinant Protein Cocktail on Human Renal Tubular Cells Survival, Proliferation, and Anti-apoptosis—Conditioned Medium vs. Recombinant Protein Cocktail

**FIGURE 4:**
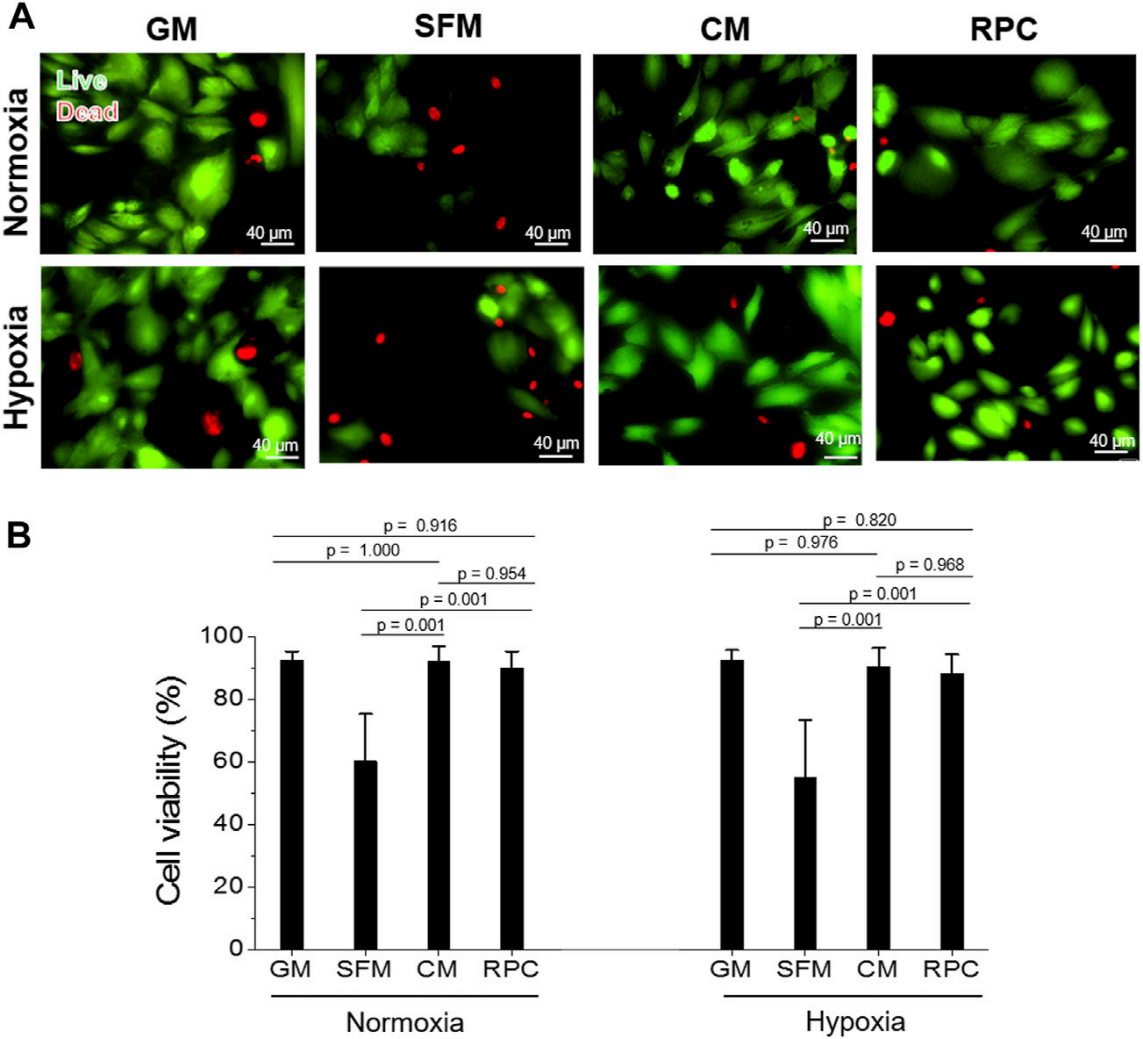
hRC survival—Conditioned medium (CM) vs. RPC. hRC culture in GM, SFM, CM, or RPC for 3 days under normoxic and hypoxic conditions. **(A)** Live (green)/dead (red) assay images. **(B)** Quantification of cell viability (%) (*n* = 8—9 per group, one-way ANOVA and Tukey’s test). Scale bars: 40 µM.

**FIGURE 5:**
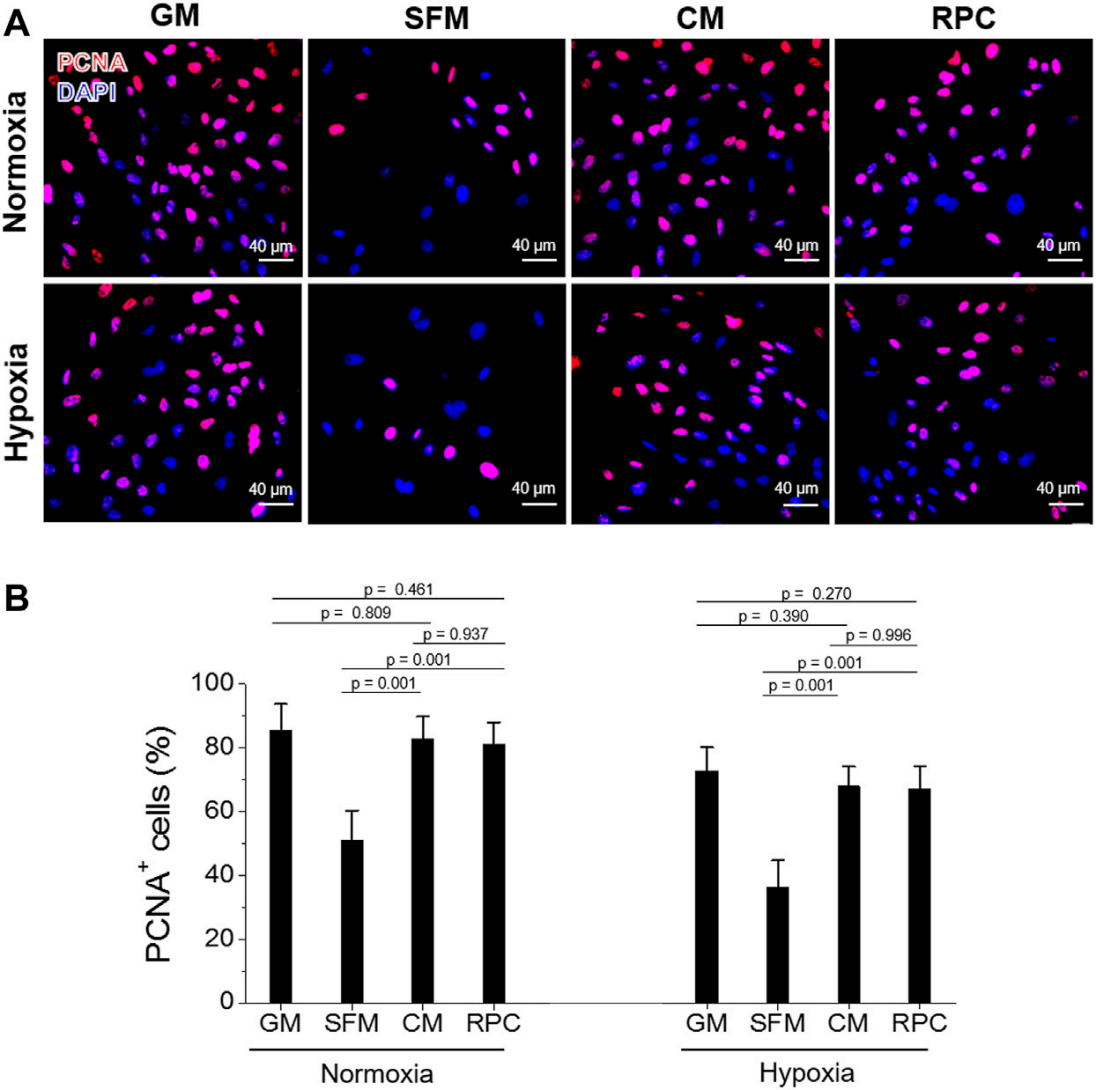
hRC proliferation—CM vs. RPC. hRC culture in GM, SFM, CM or RPC for 3 days under normoxic and hypoxic conditions. **(A)** Immunofluorescence for PCNA (red)/DAPI (blue). **(B)** Quantification of PCNA^+^ cells (%) (*n* = 10—15 per group, one-way ANOVA and Tukey’s test). Scale bars: 40 µM.

**FIGURE 6:**
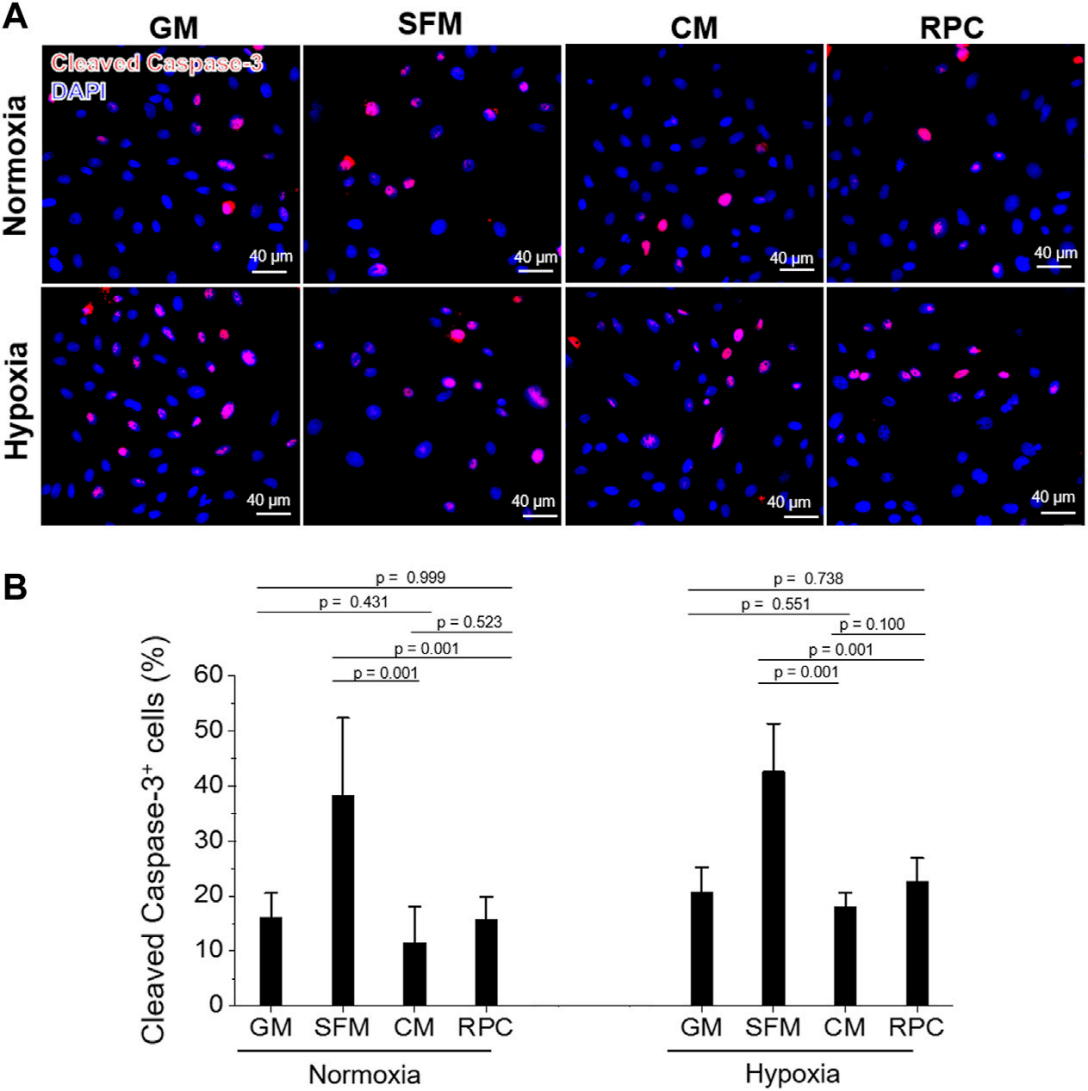
hRC apoptosis—CM vs. RPC. hRC culture in GM, SFM, CM or RPC for 3 days under normoxic and hypoxic conditions. **(A)** Immunofluorescence for cleaved caspase-3 (red)/DAPI (blue). **(B)** Quantification of cleaved caspase-3^+^ cells (%) (*n* = 14—15 per group, one-way ANOVA and Tukey’s test). Scale bars: 40 µM.

To investigate the feasibility of using the RPC as a substitute for CM, human hRCs were cultured in CM and RPC under normoxic and hypoxic conditions. Cell viability (the number of live cells per the number of total cells, %) was calculated using the live/dead assay images (*n* = 8—9 per group, one-way ANOVA, and Tukey’s test) (Figure 4). Cell proliferation and apoptosis were evaluated using immunofluorescent staining for PCNA (*n* = 10—15 per group, one-way ANOVA and Tukey’s test) (Figure 5) and cleaved caspase-3 (*n* = 14—15 per group, one-way ANOVA and Tukey’s test) (Figure 6), respectively. Both CM and RPC showed significantly increased hRC viability and proliferation compared with the SFM group in both normoxic and hypoxic conditions (*p* = 0.001). The RPC showed similar effects as CM and GM in hRC survival (*p* = 0.954 with CM and *p* = 0.916 with GM in normoxia, *p* = 0.968 with CM and *p* = 0.820 with GM in hypoxia) and proliferation (*p* = 0.937 with CM and *p* = 0.461 with GM in normoxia, *p* = 0.996 with CM and *p* = 0.270 with GM in hypoxia) (Figures 4, 5). The CM and RPC groups showed reduced cell apoptosis compared with the SFM group (*p* = 0.001); however, the RPC group was not significantly different from the GM and CM groups in hRC apoptosis (*p* = 0.523 with CM and *p* = 0.999 with GM in normoxia, *p* = 0.100 with CM and *p* = 0.738 with GM in hypoxia) (Figure 6). Additionally, human PCNA ELISA assay results showed a similar trend in the hypoxic condition (0.1% O_2_). The PCNA protein level of the RPC group was significantly higher than that of the SFM group (*p* = 0.001), but not statistically different from that of the CM group (*p* = 0.672) (*n* = 4, per group, one-way ANOVA and Tukey’s test) (Supplementary Figure S3). The AQP-1 staining results demonstrated that the phenotype of the hRC was not changed in the culture with RPC (*n* = 15 per group, one-way ANOVA and Tukey’s test, *p* = 0.700 with GM in normoxia, *p* = 0.667 with GM in hypoxia) (Supplementary Figure S4). These results indicate that the RPC treatment effectively supports hRC viability and proliferation and prevents apoptosis in normoxic and hypoxic conditions. The treatment effect of RPC is comparable to that of the CM and GM groups.

### Recovery of Renal Function by Intrarenal Delivery of Recombinant Protein Cocktail

**FIGURE 7:**
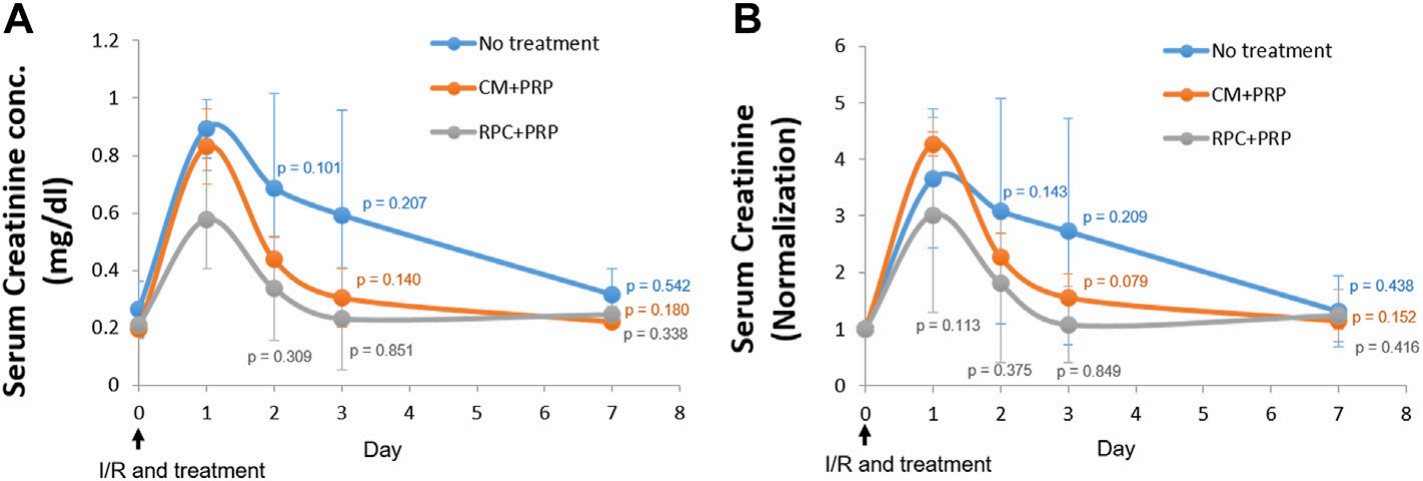
Improvement of renal function by intrarenal delivery of RPC with platelet-rich plasma (PRP) in an ischemia/reperfusion (I/R)-induced acute kidney injury (AKI) model in rats. Immediately after I/R injury, saline (no treatment), CM encapsulated in PRP (CM + PRP), or RPC encapsulated in PRP (RPC + PRP) was injected into the kidneys. Blood samples were collected at day 0, 1, 2, 3, and 7 post-I/R and injections and serum creatine levels were measured. **(A)** Serum creatinine concentration (mg/dl) and **(B)** normalized serum creatinine value (Serum creatinine concentration at each time point is divided by that at day 0) (*n* = 3 per group and time point, two-way ANOVA and Tukey’s test).

The therapeutic potential of RPC delivery using PRP in kidney disease was investigated in the I/R-induced AKI model in rats. Immediately after induction of bilateral I/R injury to the kidneys, saline (no treatment), CM encapsulated in PRP (CM + PRP), or RPC encapsulated in PRP (RPC + PRP) was injected into the kidneys. Blood samples were collected at 0 (pre-injury and injections), 1, 2, 3, and 7 days post-injury and injections, and serum creatinine levels were measured. The serum creatinine levels were expressed as serum creatinine concentration (mg/dl) (Figure 7A) and normalized serum creatinine value (Figure 7B), which were calculated by dividing the serum creatinine concentration at each time point to that at day 0 (*n* = 3 per group and time point, two-way ANOVA and Tukey’s test). In all experimental groups, serum creatinine concentration and normalized value were increased at day 1, followed by a gradual decrease until day 7. In addition, the serum creatinine levels in all experimental groups at day 3 and day 7 showed no significant differences compared to those at day 0 **(** Figure 7B **)**. Although there were no significant differences between the groups at each time point, the CM + PRP and RPC + PRP groups maintained lower serum creatinine levels than the no-treatment group up to day 7. Notably, the normalized serum creatinine value of the RPC + PRP was not significantly different between the time points, and only the RPC + PRP group showed no significant difference in normalized serum creatinine value at day 1 (*p* = 0.113) (Figure 7B). These data indicate that the RPC + PRP effectively prevents the increase of serum creatinine levels after the I/R injury.

### Amelioration of Renal Tubular Injury

**FIGURE 8:**
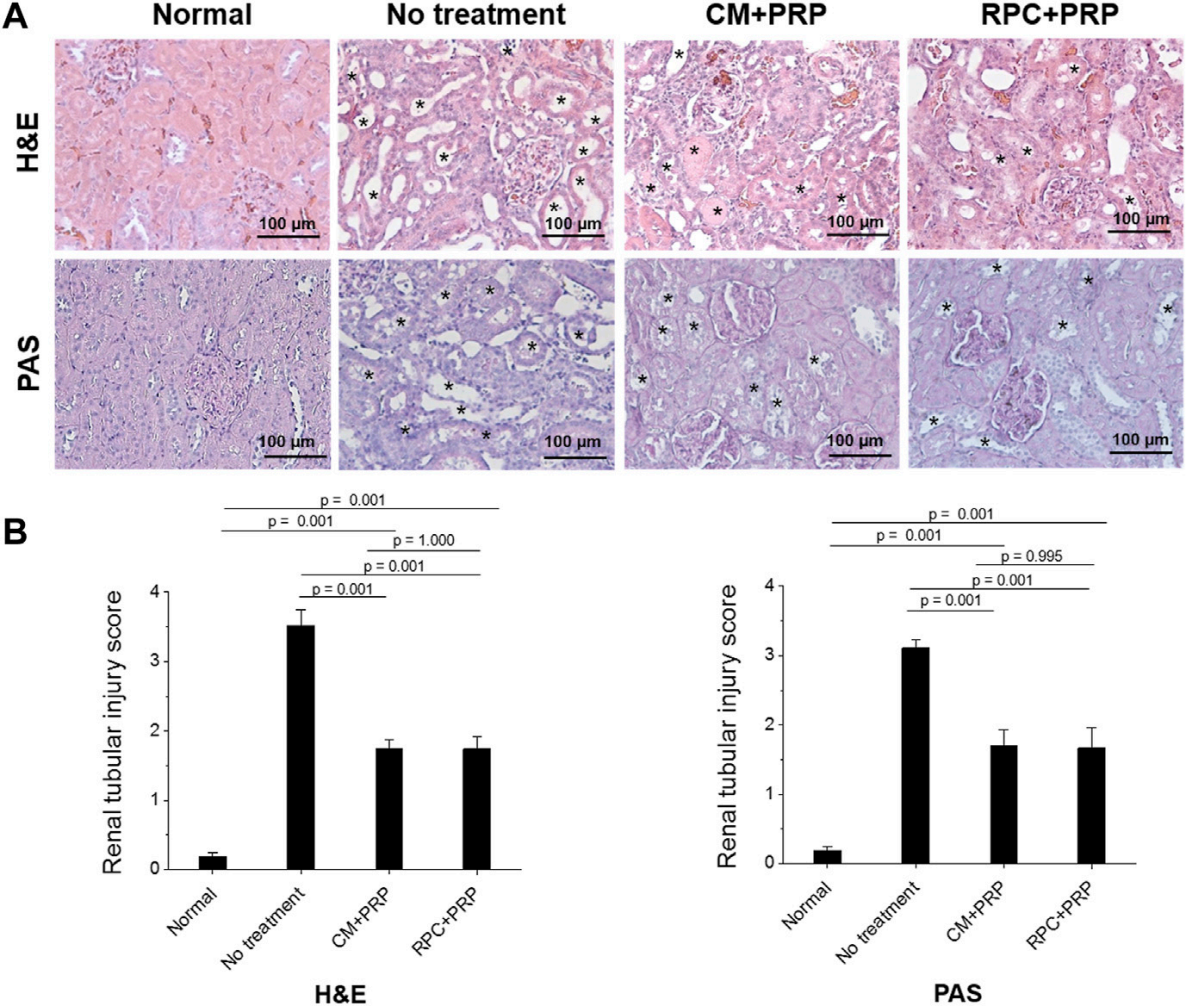
Amelioration of renal tubular damages by the treatment of RPC with PRP. **(A)** Representative images with Hematoxylin and Eosin (H&E) and Periodic acid-Schiff (PAS) staining of normal (age-matched control), no treatment, CM + PRP, and RPC + PRP groups at 7 days post-I/R and injections (asterisks: acute tubular injury). **(B)** Renal tubular injury scores obtained from H&E and PAS stained tissues (*n* = 3 per group and time point, nine random fields per sample (total 27 fields per group), one-way ANOVA and Tukey’s test). Scale bars: 100 µM.

The effect of the RPC delivery with PRP on preventing renal tubular damage was evaluated using histological analyses of the kidney tissue. H&E and PAS staining images of the CM + PRP and RPC + PRP groups showed a significant reduction in tubular dilatation, detachment of tubular epithelial cells, and condensation of tubular nuclei compared with the no treatment group (Figure 8A). In addition, reduced brush border loss in the proximal tubules was observed in the PAS staining images. Based on the H&E and PAS staining images, the level of renal tubular damage of each group was measured using the renal tubular injury scoring system (*n* = 3 per group, nine random fields per sample (total 27 fields per group), one-way ANOVA, and Tukey’s test). The RPC + PRP group showed a significantly decreased renal tubular injury score compared with the no treatment group (*p* = 0.001 in H&E and PAS staining), showing no significant difference with the CM + PRP group (*p* = 1.000 in H&E, *p* = 0.995 in PAS) (Figure 8B). These results indicate that the intrarenal delivery of RPC with PRP can ameliorate renal tubular damages at the same level as CM delivery in the I/R-induced AKI model.

### Renal Cells Proliferation and Anti-apoptosis

**FIGURE 9:**
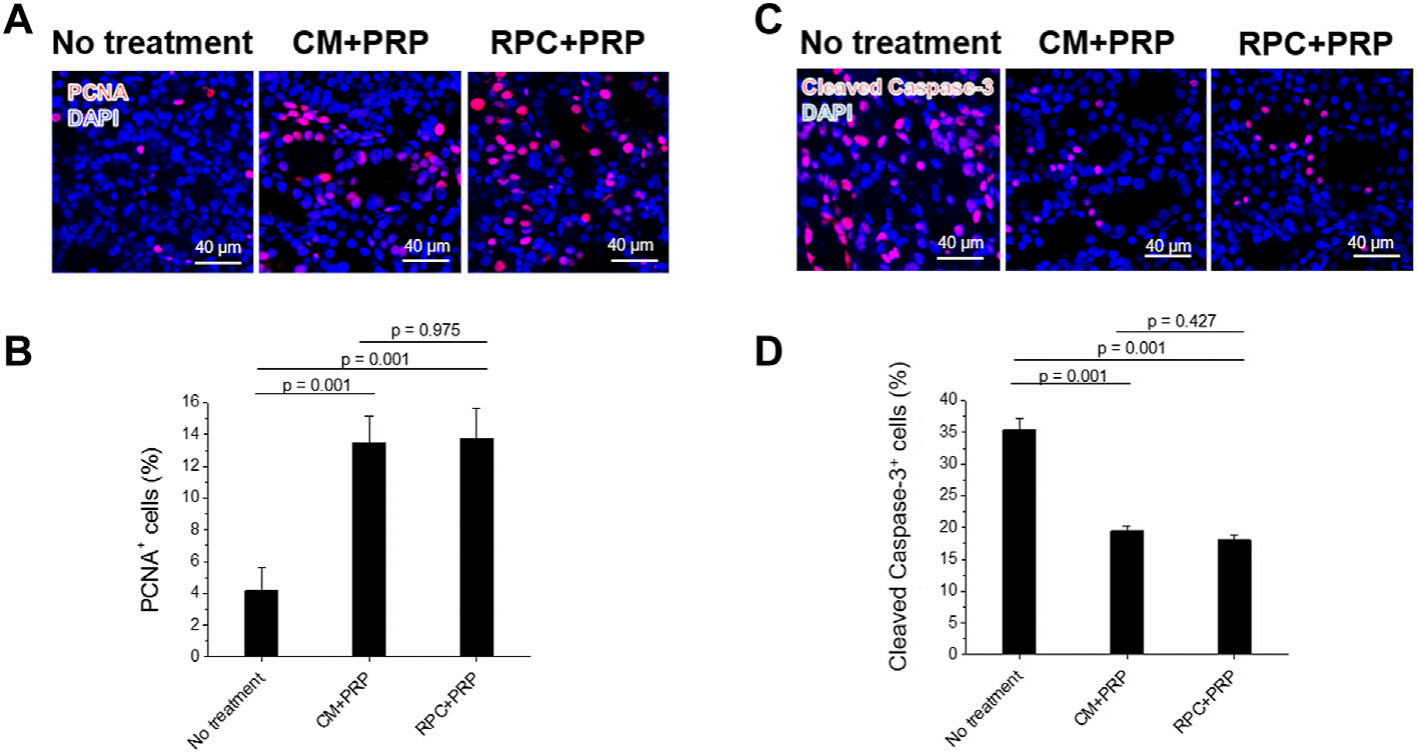
Increased cell proliferation and reduced apoptosis by the treatment of RPC with PRP. **(A,B)** Immunofluorescence for PCNA (red)/DAPI (blue) **(A)** and cleaved caspase-3 (red)/DAPI (blue) **(B)** of no treatment, CM + PRP, and RPC + PRP groups at 7 days post-I/R and injections. **(C,D)** Quantification of PCNA^+^ cells (%) **(C)** and cleaved caspase-3^+^ cells (%) **(D)** (*n* = 3 per group, five random fields per sample (total 15 fields per group), one-way ANOVA and Tukey’s test). Scale bars: 40 µM.

Renal cell proliferation and apoptosis of each group were determined using PCNA and cleaved caspase-3 stainings (*n* = 3 per group, five random fields per sample (total 15 fields per group), one-way ANOVA, and Tukey’s test) (Figure 9). The CM + PRP and RPC + PRP groups showed significantly increased PCNA^+^ cells (%) and decreased cleaved caspase-3^+^ cells (%) compared with the no treatment group (*p* = 0.001). There was no significant difference between the CM + PRP and RPC + PRP groups in PCNA^+^ cells (%) (*p* = 0.975) and cleaved caspase-3^+^ cells (%) (*p* = 0.427). These results demonstrate that the RPC delivery has the same proliferative and anti-apoptotic effects as CM delivery in the I/R injury-induced kidney.

## Discussion

Over the last decade, cell therapy has been introduced as a regenerative tool to treat damaged tissues, including AKI. Several studies showed significant renoprotective effects of the cell therapies (Yamaleyeva et al., 2012; Stenvinkel et al., 2016; Kim et al., 2020). Although promising, the therapeutic effect of the cell therapies is mainly attributed to the secretion and release of trophic molecules (secretomes) of the transplanted cells, not their capability of engrafting and differentiation into the renal cell types in injured sites (Srijaya et al., 2014; Lukomska et al., 2019; Yim et al., 2019). Such a paracrine action has the therapeutic potential for cell-free treatments using secretomes. Therefore, the use of cell-derived secretomes in CM as a whole or its components has emerged as a therapeutic option for kidney diseases.

The reno-protective effects of stem cell-derived secretomes have been investigated as a potential active pharmaceutical component in the last decade, demonstrating that they promoted tubular recovery in kidney disease models. For instance, whole secretomes as a form of CM have been used to treat kidney diseases. For example, intraperitoneal or intravenous injections of MSC (da Silva et al., 2015; Abedi et al., 2016; Liu et al., 2018), hPSC (Yim et al., 2019), ESC (van Koppen et al., 2012), or iPSC (Tarng et al., 2016) -derived CM showed a decreased progression of renal damages and improved renal function in animal models of kidney diseases. The therapeutic efficacy was likely mediated by reduced anti-apoptosis and increased renal cell proliferation in these studies.

While the approaches using the whole secretomes as a form of CM have been promising for renal treatment, many challenges remain to be addressed before clinical translation. Further study is required to better characterize the secretome and define which factors are responsible for its therapeutic effects. Additionally, not all secretomes in CM are beneficial, and CM can contain proteins released from dead cells or cells undergoing apoptosis (Skalnikova et al., 2011; Liang et al., 2014). The use of well-defined secretomes would be beneficial for clinical use to minimize safety and regulatory concerns. The CM production in quantities sufficient for clinical administration through a large-scale and long-term cell culture is also challenging. Furthermore, the qualities and quantities of secretomes in CM can vary depending on the cell culture conditions (Pawitan, 2014). By its nature, the complexity of the secreted factors makes it difficult to obtain consistent results in pre-clinical and clinical studies.

To address the challenges raised by the secretomes/CM therapies, our current study utilizes the combination of recombinant proteins (RPC: follistatin, VEGF, HGF, uPAR, ANGPTL4). These five proteins were selected by identifying the highly expressed trophic factors that promote kidney repair in the hPSC-CM through the proteomics study. The five recombinant proteins used in this study were selected with the following criteria—effective proteins for kidney repair and regeneration, and their concentration is higher than the physiological concentration. The proteins effective for kidney repair but lower than physiological concentration and inhibitory proteins were excluded. The secretome profile and the concentration of the five proteins were similar to those obtained in our previous study (Yim et al., 2019). Using the RPC provides key advantages over the whole secretome/CM therapies. Therapeutic agents are a combination of commercialized recombinant proteins. The use of defined composition and dosage of the RPC can make the quality control, regulation, and scale-up processes of the product more straightforward and efficient but also achieve the consistency of therapeutic outcomes, which is a crucial hurdle in translating secretomes into a clinically useful product. Our cell-free strategy eliminates a long-term and mass cell culture process, thus saving time and resources. The treatment modality using the RPC as a ready-to-use and off-the-shelf product could be immediately available for treatment when desired, enabling their application to acute conditions such as AKI.

Each protein in the RPC has been reported to prevent apoptosis, promote the proliferation of renal cells *in vitro* and *in vivo*, and attenuate renal injury and promote repair of damaged kidneys. For instance, follistatin reduces apoptosis of renal tubules and promotes cell proliferation *in vitro* and *in vivo* by inhibiting the activity of Activin, which prohibits renal tubular cell regeneration (Maeshima et al., 2001; Fang et al., 2016; Leonhard et al., 2016). HGF promotes survival, prevents apoptosis of tubular epithelial cells *in vitro* and *in vivo*, and ameliorates initial injury after I/R by protecting renal tubules from apoptosis. HGF inactivates Bad, a pro-apoptotic protein, via the phosphoinositide (PI) 3-kinase/Akt pathway, while it induces expression of anti-apoptotic Bcl-xL (Miller et al., 1994; Liu et al., 1998; Liu, 1999; Zhou D. et al., 2013; An et al., 2020). VEGF promotes the survival and proliferation of renal epithelial cells and kidney vascularization by upregulating VEGFR-2 mediated signaling cascade, including PI3K/Akt pathway (Villegas et al., 2005; Estrada et al., 2019; Fujii et al., 2019). Although the mechanisms of uPAR and ANGPTL4 for renal regeneration followed by acute injury are still widely studied, their renoprotective effects have been reported. uPAR attenuates the fibrogenic response to renal injury by degrading pro-fibrotic molecules such as PAI-1, reducing renal cell apoptosis, and promoting angiogenesis (Eddy, 2002; Zhang et al., 2003a; Zhang et al., 2003b; Ma and Fogo, 2009). ANGPTL4 reduces renal injury and proteinuria by binding to glomerular endothelial ανβ5 integrin and inactivating LPL activity and promotes angiogenesis in ischemia (Le Jan et al., 2003; Chugh et al., 2014; Clement et al., 2014). In addition, several studies demonstrated that the antagonists or inhibitors of ANGPTL4, follistatin, HGF, uPAR, and VEGF receptors aggravate the renal damage in AKI animal models (Eddy, 2002; Zhang et al., 2003a; Ma and Fogo, 2009; Zhou D. et al., 2013; Chugh et al., 2014; Clement et al., 2014; Fang et al., 2016; Leonhard et al., 2016; Estrada et al., 2019; Fujii et al., 2019; An et al., 2020; Mohan and Herrmann, 2022).

The results obtained in this study also showed pro-proliferative and/or anti-apoptotic effects in single proteins *in vitro*. ANGPTL4, HGF, uPAR, and VEGF significantly increased hRC proliferation and decreased apoptosis compared with SFM. Although follistatin did not significantly increase hRC proliferation compared with SFM in this experimental condition, its role in preventing hRC apoptosis was confirmed. The *in vivo* reno-protective effects of these proteins need to be investigated in subsequent studies in the animal model of AKI.

This study investigated the pro-proliferative and anti-apoptotic effects of a combination of five proteins (RPC) on hRC compared with individual proteins *in vitro*. The RPC treatment increased hRC proliferation and decreased apoptosis compared with ANGPTL4, follistatin, HGF, and uPAR. The RPC showed a higher hRC proliferation effect than VEGF. Only the RPC showed the proliferative and anti-apoptotic effects in both normoxic and hypoxic conditions. In addition, the RPC had similar effects as GM (positive control), but follistatin did not. Remarkably, the cell viability and pro-proliferative and anti-apoptotic effects of RPC were not statistically different from those of CM *in vitro* and *in vivo*. Therefore, it is not a single factor that exerts a therapeutic benefit but the combination of five proteins and their complex interplay that results in the desired reno-protective effects. The mechanism of action on RPC in kidney repair at the molecular level, including receptors for the five proteins, needs to be further investigated in future studies.

We used the PRP hydrogel system for efficient delivery of the RPC into the damaged kidney. While the CM and RPC themselves are an effective means to treat kidney diseases, solubilized factors can undergo rapid diffusion and lose their biological activities within a short time following administration *in vivo*, which is another limitation in translating into the clinic (Xing et al., 2014; Abedi et al., 2016). The efficient delivery system of secretome factors is necessary to achieve improved therapeutic effects. Several synthetic hydrogel systems were used for the controlled release of secretomes, such as self-assembled multi-domain peptide nanofiber (Bakota et al., 2011; Wang et al., 2011), nanoclay (Waters et al., 2016), and nanoparticles-hydrogel composite (Wang et al., 2016). Our study utilized a PRP hydrogel-based delivery system to efficiently deliver the RPC into the injured kidney. PRP is a clinically relevant autologous hydrogel source that can be obtained from the patient’s blood and minimize immune response compared with allogeneic, xenogeneic, and synthetic materials. The safety and biocompatibility of PRP were demonstrated in several clinical studies (Patel et al., 2016; Wang et al., 2019). We demonstrated that the PRP gel system facilitates the controlled and sustained release of the hPSC-CM *in vitro* and *in vivo* (Yim et al., 2019). Release kinetics could be controlled by adjusting the concentration of PRP gels. We confirmed that the controlled delivery and sustained release of the proteins in CM using the PRP gel improved the recovery of damaged kidneys and ameliorated renal function compared with the CM delivery without PRP and the PRP alone in the I/R injury model in rats. Therefore, in this study, the PRP system is speculated to deliver the RPC efficiently, contributing to the attenuation of renal damages.

In this study, the feasibility of using the RPC encapsulated in the PRP system to treat renal injuries was investigated in the I/R injury-induced AKI model in rats. The intrarenal delivery of RPC with the PRP system could attenuate renal tubular damages by preventing apoptosis and enhancing proliferation of renal cells *in vivo*, yielding similar effects to the CM delivery. Additionally, the RPC with PRP treatment decreased the serum creatinine level and facilitated the rapid restoration of renal function. The serum creatinine concentration of the RPC delivery group was slightly increased at postoperative day 1, but the normalized serum creatinine level was not significantly increased compared with the pre-operative serum creatinine level (day 0 baseline). As observed in the histological and functional results, the delivery of RPC with the PRP system can attenuate the progression of renal diseases. The RPC can be a potential alternative to the CM for the treatment of AKI.

Although the strategy of using the delivery of RPC shows promising for treating kidney diseases, several issues still need to be addressed for clinical translation. This study focused on investigating the effects on renal cell survival and proliferation *in vitro* and *in vivo*. Other biological roles of the RPC beyond cell survival and proliferation need to be investigated, such as angiogenesis. The reno-protective function and effectiveness of other proteins not screened or selected in this study cannot be roled out. More comprehensive screening of secretomes is needed. Further investigation on the selection and combination of proteins is needed to maximize therapeutic outcomes. In this pilot study, we used recombinant human proteins derived from insects or mammalian cells. For future clinical trials, the modification with glycosylation on the proteins as a therapeutic product during a manufacturing process and the impact on the safety and clinical efficacy need to be investigated. Optimization of the delivery system would be necessary to consistently supply the regenerative proteins to target sites. As noted above, the PRP gels provide several advantages over other delivery vehicles for the clinically applicable autologous sources. Because of its nature, however, an issue associated with thrombus formation was raised when high-dose PRP was administered (Martin-Sole et al., 2016). Alternative clinically applicable delivery vehicles such as collagen and fibrinogen may be tested. Future studies will investigate the host responses, including inflammatory and immune responses, and conduct various functional analyses to assess the therapeutic effects (e.g., blood urea nitrogen, urine output) in a large-scale pre-clinical study using immunocompetent animal models.

## Conclusion

In conclusion, the delivery of RPC can support renal cell survival and proliferation *in vitro* and *in vivo*, resulting in the attenuation of renal tubular damages and amelioration of renal function in the I/R-induced AKI model in rats. With further advances, the strategy of RPC delivery could be an effective therapeutic approach for repairing kidney diseases. Our strategy may provide a novel solution to many challenges associated with kidney regeneration resulting from the lack of suitable regenerative medicine therapies and offer an off-the-shelf product for structural and functional recovery from kidney damages.

## Data Availability

The raw data supporting the conclusions of this article will be made available by the authors, without undue reservation.
