# Supplementary material for: The Delivery of the Recombinant Protein Cocktail Identified by Stem Cell-Derived Secretome Analysis Accelerates Kidney Repair After Renal Ischemia-Reperfusion Injury
Source: Front Bioeng Biotechnol. 2022 May 11;10:848679. doi: 10.3389/fbioe.2022.848679 (PMC9130839; doi:10.3389/fbioe.2022.848679)
Supplement: Supplementary file 1 [file DataSheet1.PDF]

Supplementary Material

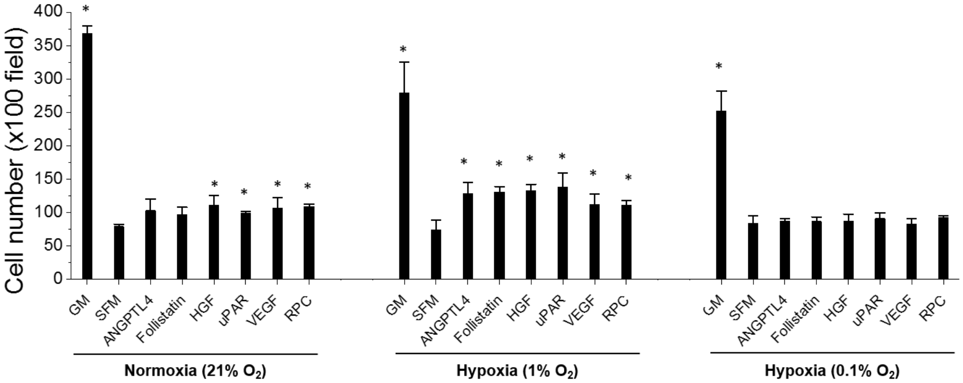

Supplementary Figure 1. Human primary renal cell (hRC) proliferation - Single protein vs. combinatorial proteins. Quantification of cell numbers (×100 magnification) treated with growth medium (GM), serum free medium (SFM), single protein (ANGPTL4, follistatin, HGF, uPAR, or VEGF) or their combination (recombinant protein cocktail (RPC)) for 3-day culture under normoxic (21% O_2) and hypoxic (1% O_2 or 0.1% O_2) conditions ($n = 3$ per group, ANOVA and Tukey's test, $^*P < 0.038$ with SFM). Data presented as mean ± standard deviation.

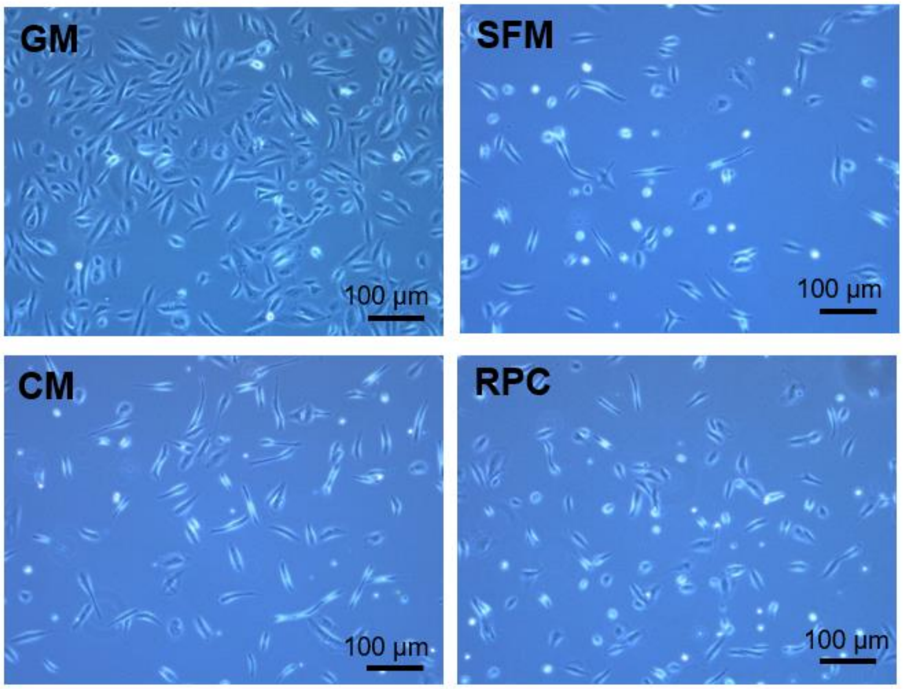

Supplementary Figure 2. Morphology of hRCs after treatment of GM, SFM, conditioned medium (CM) or RPC for 3-day culture under normoxic condition (21% O_2). Scale bars: 100 µm.

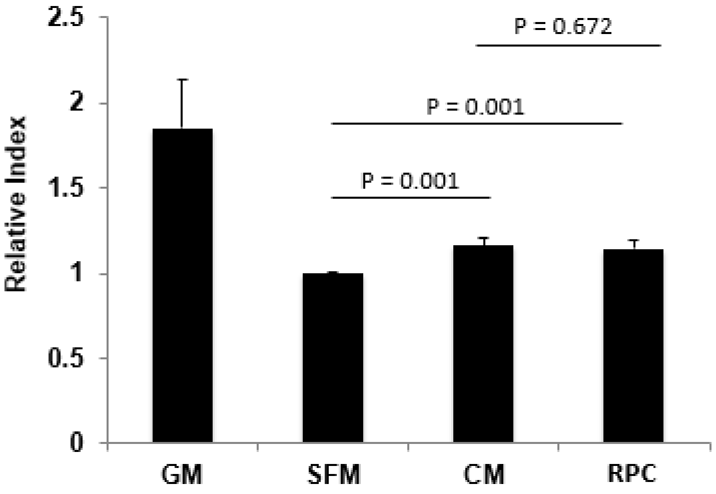

Supplementary Figure 3. hRC proliferation by treatment of RPC. hRC culture for 3 days under hypoxic condition (0.1%, O_2). The proliferating cell nuclear antigen (PCNA) expression of each group is measured using human PCNA ELISA and the value is normalized by that of SFM (*n* = 4 per group, ANOVA and Tukey's test).

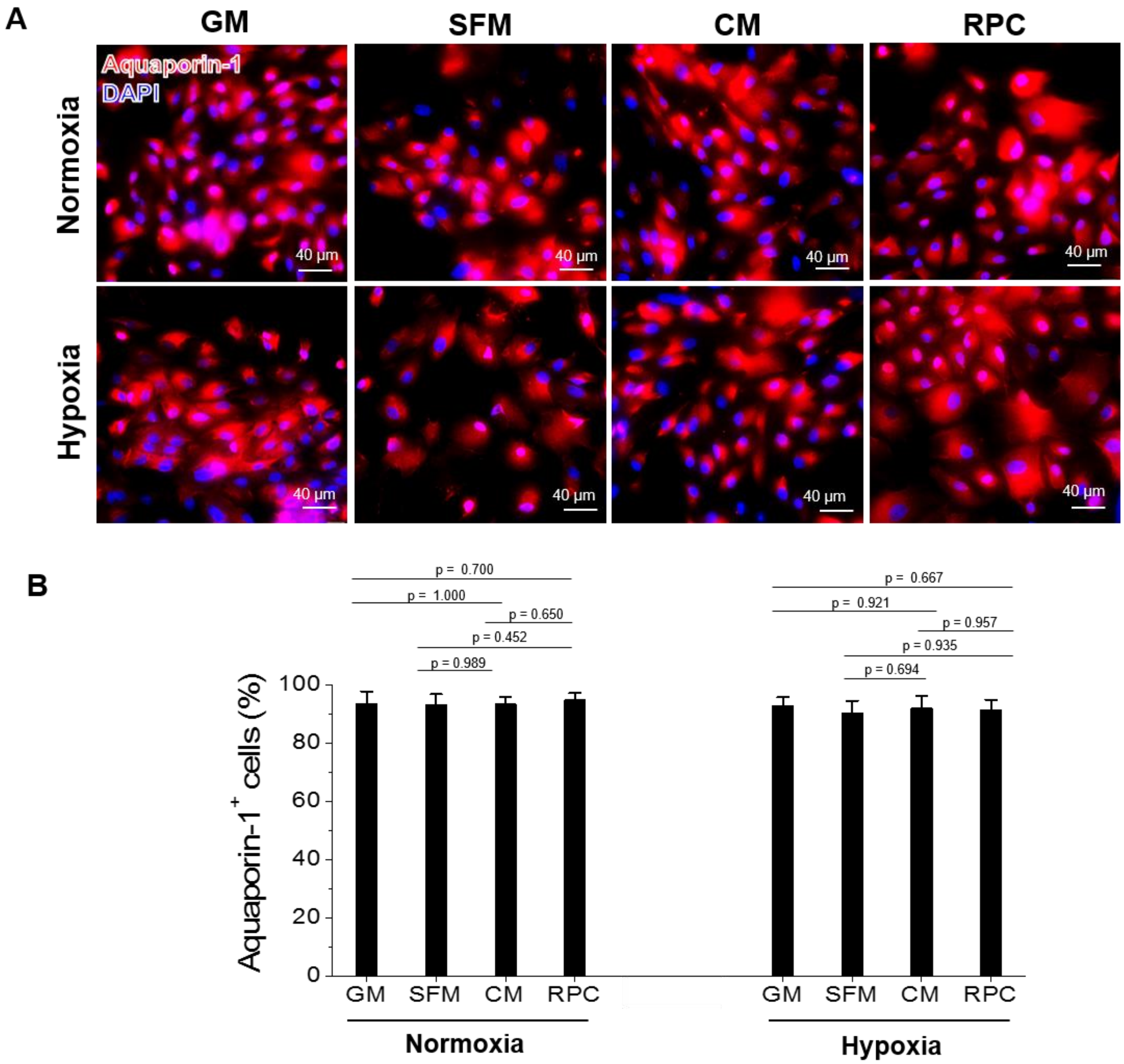

Supplementary Figure 4. hRC phenotype. hRC culture in GM, SFM, CM or RPC for 3-days under normoxic and hypoxic conditions. **(A)** Immunofluorescence for aquaporin-1 (red)/DAPI (blue). **(B)** Quantification of aquaporin-1^+ cells (%) (n = 15 per group, one-way ANOVA and Tukey's test). Scale bars: 40 µm.
